# Effects of Thermal Annealing on Femtosecond Laser Micromachined Glass Surfaces

**DOI:** 10.3390/mi12020180

**Published:** 2021-02-11

**Authors:** Federico Sala, Petra Paié, Rebeca Martínez Vázquez, Roberto Osellame, Francesca Bragheri

**Affiliations:** 1 Department of Physics, Politecnico di Milano, Piazza Leonardo da Vinci 32, 20133 Milano, Italy; federico.sala@polimi.it (F.S.); roberto.osellame@cnr.it (R.O.); 2 Istituto di Fotonica e Nanotecnologie, CNR, Piazza Leonardo da Vinci 32, 20133 Milano, Italy; rebeca.martinez@polimi.it (R.M.V.); francesca.bragheri@ifn.cnr.it (F.B.)

**Keywords:** femtosecond laser micromachining, fused silica, roughness analysis, thermal annealing, integrated optics

## Abstract

Femtosecond laser micromachining (FLM) of fused silica allows for the realization of three-dimensional embedded optical elements and microchannels with micrometric feature size. The performances of these components are strongly affected by the machined surface quality and residual roughness. The polishing of 3D buried structures in glass was demonstrated using different thermal annealing processes, but precise control of the residual roughness obtained with this technique is still missing. In this work, we investigate how the FLM irradiation parameters affect surface roughness and we characterize the improvement of surface quality after thermal annealing. As a result, we achieved a strong roughness reduction, from an average value of 49 nm down to 19 nm. As a proof of concept, we studied the imaging performances of embedded mirrors before and after thermal polishing, showing the capacity to preserve a minimum feature size of the reflected image lower than 5μm. These results allow for us to push forward the capabilities of this enabling fabrication technology, and they can be used as a starting point to improve the performances of more complex optical elements, such as hollow waveguides or micro-lenses.

## 1. Introduction

Femtosecond laser micromachining (FLM) is a versatile technique that allows for the microstructuring of different types of materials. A laser source, with a pulse duration ranging from few tens to many hundreds of femtoseconds, is focused inside of a transparent material. Inside the focal volume, thanks to the high intensity, nonlinear phenomena occur, permanently modifying the substrate. It is possible to move the focal point inside the volume in the three dimensions, laser-writing the desired geometry inside the material. The most versatile class of materials that can be employed for FLM laser writing is glass. Depending on the type of glass and on the irradiation parameters, it is possible to induce different types of modifications. A first type of modification, shared by many types of glasses, is a local permanent change of the refractive index that can be used to realize waveguides and integrated photonic circuits [1]. A second type of modification, characterized by a strong birefringence, can be induced in certain glasses [2,3,4,5,6] by changing the irradiation parameters. This type of modification takes the name of *nanogratings*, and it is characterized by periodic nanostructuring of the laser track in the form of lamellae, usually oriented perpendicularly to the femtosecond laser linear polarization. These nanogratings express a highly enhanced etching rate in specific acids, like hydrofluoric acid (HF). This opens the possibility to selectively microstructure the glass, realizing embedded channels and cavities. The combination of laser irradiation and acid attack takes the name FLICE (femtosecond laser irradiation followed by chemical etching) [7]. An example of glass that presents both types of modifications, depending on the writing laser fluence [8], is fused silica. Its high transparency, mechanical robustness, and chemical inertia combined with the possibility to realize both microstructures and photonic elements make fused silica an optimal platform for the realization of integrated lab-on-chip (LOC) devices in the field of integrated photonics [1], optomechanics [9], and optofluidics [10,11].

The two main building blocks of LOCs are microchannels and micro-optics. Embedded channels are used in microfluidic applications to confine the fluids in a laminar regime and to perform many types of operations such as fluid mixing or filtration, and particle focusing, sorting, delivering, and handling [12]. With FLICE, they can be realized directly inside the volume, or they can have an open geometry, sealed afterwards. A first example of integrated optical components fabricated by FLICE is given by embedded mirrors that make use of total internal reflection (TIR) [13,14,15] or filled with metallic media [16]. A second example is the realization of focusing elements such as micro-lenses [17], hollow micro-lenses [15], filled micro-lenses [18], or diffraction-based elements [19]. These two components are highly sensitive to a specific manufacturing parameter that can strongly affect their performances: the residual surface roughness. Rough microchannel surfaces can affect the quality of the images taken when looking with a microscope inside the channel. On the other hand, the roughness strongly affects the optics performances, both in the case of light scattering, total internal reflection [20], or light confinement. As a consequence, control over the residual surface roughness is a main issue in FLICE fabrications.

From the literature, the FLICE residual roughness (root mean squared (RMS)) in the case of fused silica is around 60 nm and it strongly depends on laser irradiation polarization [21,22] and scan direction, i.e., on whether the surface is parallel or orthogonal to the writing laser propagation axis [23]. In order to improve the surface quality, several treatments have been proposed. A first example is local thermal annealing of exposed surfaces, performed with oxyhydrogen flame. This procedure causes partial melting of the most superficial layers and subsequent smoothing thanks to surface tension [17,24]. It can be combined with glass stretching [25]. This approach was used to smooth a buried microchannel, but it has a major drawback. The structures are significantly elongated along the drawing direction; thus, this technique is not suitable for complex geometries in which the shape of the microstructures should be preserved, such as in the case of integrated lenses. Another technique for local thermal annealing is CO${}_{2}$ laser selective melting [26,27,28,29], where an infrared laser source is used to locally melt the surface of an exposed or shallow-buried microchannel. Alternatively, the surfaces can be smoothed with isothermal annealing of the whole volume [30] in a furnace, with a maximum temperature just above the annealing point of the material in order to release the internal stress and to smooth superficial asperities. This approach has the advantage of being suitable for the smoothing of buried microstructures, regardless of their orientation or geometry, and is particularly appealing for optofluidic applications due to the possibility to improve the surface quality without altering the surface profile.

Starting from these previous results, in this work, we present a comprehensive study that, for the first time, combines FLM irradiation parameter optimization and thermal annealing, demonstrating the possibility to highly improve the surface quality of optical elements. We realized flat surfaces with the FLICE technique and HF etching and characterized their roughness in terms of average values and spatial features in order to optimize the laser-writing geometry. Afterwards, we define a thermal annealing procedure for surface smoothing and quantify its effects with comparative analysis. Lastly, as a proof of concept, we show the effectiveness of thermal smoothing with the realization of millimetric embedded mirrors. The analysis of reflected images acquired through a treated and untreated mirror highlights a clear surface quality improvement after the thermal annealing.

## 2. Results

### 2.1. Optimization of Laser Irradiation

In our optimization work, we decided to start from the most simple 2D surface, i.e., a flat surface, written along the laser propagation axis (z axis—see Section 4.1). Because of mounting constraints of the sample in the measurement instrumentation, we realized millimetric-sized surfaces on a paralellepiped of 2 mm by 1 mm by 1 mm.

The first step in the analysis consisted of studying the effect of slot irradiation geometry (see Section 4.1 for further details) on the final roughness. All laser irradiation parameters were chosen from previous experiments in order to guarantee a uniform etching speed at different sample depths. We realized 5 different samples by varying the slot thickness, the vertical pitch between the irradiation lines, and the orientation with respect to the laser polarization. All details are reported in Table 1.

**Figure 1:**
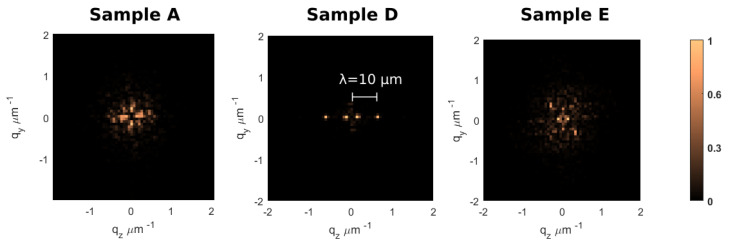
Two-dimensional power spectral densities (PSD) of three samples before thermal annealing. Sample A was written with standard parameters. Sample D had the coarser z spacing (dz). Sample E was written with standard parameters but with parallel linear polarization. Each plot is normalized with respect to its maximum.

The measured root mean squared (RMS or $S_{q}$) roughnesses are reported in the table. The results are in line with the literature, and it is confirmed that a polarization orthogonal with respect to the sample translation direction gives a lower S${}_{q}$ value. Interesting considerations could be inferred when looking at the spatial frequency components of the profiles, as evident in Figure 1, where the 2D power spectral densities (PSDs${}^{2D}$) of three samples are shown. The data of sample A present a radially symmetrical distribution, representing a randomly distributed height profile. On the other hand, sample D, written with a coarser spacing of the laser lines along z direction, shows a sharp peak at the spatial frequency corresponding to a spatial wavelength of 10μm. It has to be underlined that these peaks correspond to the sample z spacing; thus, it can be linked to periodic modulation of the surface as reminiscence of the irradiation pattern. A similar effect was seen also in sample E (not reported) even with a lower contrast. A secondary peak, corresponding to a spatial wavelength of 50μm, is also visible, but it is present also in all samples and it has been attributed to the Fourier Transform windowing effect. Lastly, sample E, written parallel to the laser polarization, presents a random distribution profile, coherent with the lack of a preferential etching direction and thus with a more homogeneous acid attack [21].

Analysis of the spatial distribution of the roughness suggests that the z spacing is fundamental in order to control surface characteristics. Indeed, a too coarse pitch may lead to a non-uniform superposition of laser tracks along z direction, even if the spacing is smaller than the dimension of the modified material spot (a typical z dimension of a laser track corresponds to 20–30 μm depending on the depth in the sample and on the focusing objective). As a consequence, a periodic texturing arises along the z direction. This effect can be explained by looking at the evolution of the etching track during time. After the removal of the irradiated volume, the single laser line enlarges isotropically around its axis, until it coalesces with the adjacent tracks in a more random geometry. This type of phenomena is known in lithography, studying the evolution of surface scratches when exposed to HF [31]. Maintaining the etching time constant, the samples with a finer irradiation pattern develop a more random surface texture with respect to those with a coarser spacing. In order to mitigate the contribution of high-frequency components, one can increase the etching time [31] at the cost of a higher deformation of the original irradiated geometry [7]. In view of these considerations, we chose 5μm z spacing as our upper limit. Once we guaranteed a sufficient superposition of the irradiation lines, the roughness distribution became more random, with a PSD${}^{2D}$ profile similar to a single pole exponential decay, typical of mechanically grinded surfaces [32].

### 2.2. Thermal Annealing Characterization

**Figure 2:**
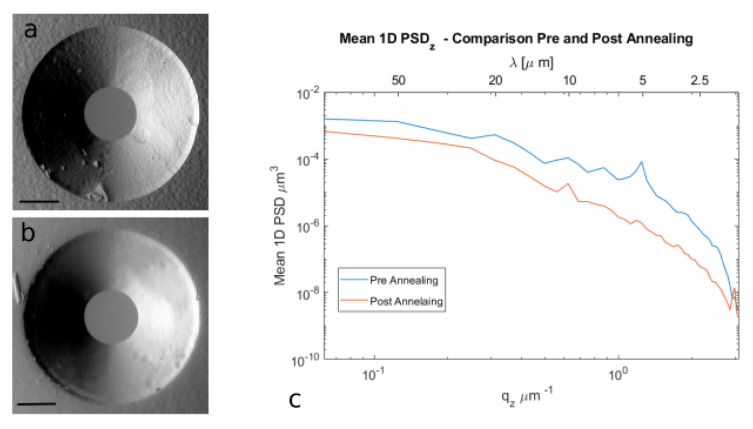
Thermal annealing effect on surface roughness. SEM images of two microstructured cones before (**a**) and after (**b**) thermal treatment. Scale bars correspond to 50μm; (**c**) comparison of sample C 1D PSD${}_{z}$ before and after the thermal treatment, reported in logaritmic scale.

The glass samples were treated with isothermal annealing with the recipe described in Section 4. The procedure was optimized to reduce the possibility of generation of cracks on the exposed surface. Several test samples were inspected at the optical microscope and scanning electron microscopy (SEM) in order to qualitatively evaluate the surface quality and to optimize the annealing procedure. During this optimization, we used test samples with a parallelepiped shape, such as the one presented before, and FLICE-microstructured samples with surface-exposed structures such as wedges or cones. An example of these is reported in Figure 2. In subfigures (a) and (b), the SEM image of a cone with a central circular microchannel is reported before and after the thermal annealing. The reduction in surface roughness is qualitatively clear.

We studied the effects of the treatment on three samples, samples B and C, that present higher and lower roughnesses for perpendicular polarization, and sample E, written with parallel polarization. The three samples were analyzed a second time with the stylus profilometer, focusing on an area in the same position as the previous measurement. We obtained a reduced $S_{q}$ in all three cases, 25 nm, 20 nm, and 12 nm, for samples B, C, and E, respectively, starting from values of 57 nm, 28 nm, and 63 nm. In Figure 2c, the 1D PSD of sample C is shown, obtained by averaging the PSD${}^{2D}$ along y axis. It is clear that the annealing reduced the RMS roughness (that corresponds to the area under the graph) and that it had a higher influence on the high-frequency components, i.e., on the right-handed part of the plot. This is coherent with the qualitative model presented for other thermal treatments [17], stating that only the first layers of the surface undergo softening or partial melting, whereas the form, i.e., the low-frequency components, is less affected. This guarantees better preservation of the microstructured profile during the smoothing process.

These results show that isothermal annealing can actually improve the surface roughness of surfaces of embedded optical elements, smoothing those frequency components that are responsible for undesired scattering and that cause minor modifications on the form of the element.

As a proof of concept of the efficiency of the thermal smoothing, we realized and tested an embedded flat mirror. This mirror consists of hollow slot along the z axis realized with the same irradiation geometry shown before. In order to appreciate the removal of surface patterning after the smoothing, we chose to use the sample C irradiation parameters. The slot was oriented at 45${}^{\circ }$ with respect to the glass sample facets and was used as a total internal reflection-based mirror. The sample was characterized before and after thermal annealing in order to evaluate the changes in the optical performances.

**Figure 3:**
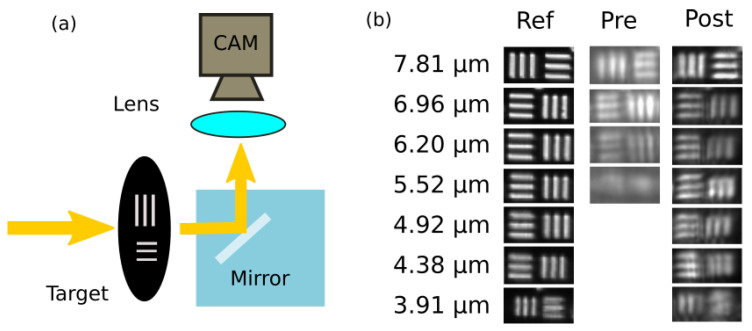
Optical performances comparison: (**a**) scheme of the experiment; (**b**) comparison of USAF-1951 target images, between the reference (Ref), not-treated mirror (Pre), and smoothed mirror (Post). A blurring effect is evident, induced by the residual roughness in the images from not-treated mirrors that contribute to increasing the background and to reducing the contrast.

The scheme of this experiment, together with a drawing of the mirror, is reported in Figure 3a. Collimated light was used to illuminate a target. The transmitted light, once reflected by the embedded mirror, was focused by a microscope objective on a camera. An image of the same target, with the same objective and with a standard silver mirror, was taken as a reference. The three images (reference, not-annealed mirror, and annealed mirror) are reported in Figure 3b. In the case of the reference optical system, we were able to clearly distinguish up to the smallest feature size of the target in analysis (element 7.6 of the USAF-1951 target, corresponding to 2.19μm). As a consequence, any degradation of the images is imputable to the surface quality of the mirrors. In order to determine the maximum resolution, we used Raylegh criterion: the difference in intensity between two peaks and the dip in between should be greater than 20% of the peak. In the case of the not-treated mirror, we obtained a vertical resolution of 6.96μm and a horizontal one of 6.20μm. In the case of the annealed one, we obtained an improved result of 4.38μm along the vertical direction and 4.92μm along the horizontal.

These results show how the roughness reduction after thermal annealing could be beneficial for the performances of embedded total internal reflection mirrors. The reduction in high-frequency components of the PSD reduces the undesired distortion introduced by the mirror. The difference in resolution between horizontal and vertical directions, i.e., y and z axes of the mirror surface, is coherent with the results on the PSD${}^{2D}$.

## 3. Discussion

In this work, we analyzed the effect of irradiation geometry optimization and isothermal annealing on the surface quality of micro-structures realized with FLM and HF etching.

First, we verified the results reported in the literature, $S_{q}=60-30$ nm, over an area of 100μm by 100μm per sample. Analysis of the PSD${}^{2D}$ shows that increasing the spacing between adjacent irradiation lines could cause periodic patterning on the surface. In our case, a distance grater or equal to 5μm causes high-frequency peaks in the PSD${}^{2D}$. If the spacing between irradiation lines is sufficiently fine, the PSD${}^{2D}$ presents a random-like distribution, both in the case of parallel and perpendicular laser polarizations.

We quantitatively measured the effect of isothermal annealing on the fused silica etched surfaces, characterizing the spatial components that contribute to roughness. Thanks to this analysis, we verified the improvements in the surface quality and the beneficial effect on the high-frequency components of the PSD. The resulting surfaces present an $S_{q}$ ranging from 25μm to 12μm. We verified this improvement by analyzing the optical performances of two flat embedded TIR mirrors, with and without thermal annealing. We showed that the untreated mirrors provide a resolution around 6–7 μm, depending on the axis of analysis. An element such as this could still be used as a low-quality mirror or as spatial filter, but it is not suitable for most photonic applications. On the other hand, we verified that including the smoothed mirror inside an imaging system guarantees a resolution of around 4.5μm, sufficient for the detection of cell-like samples in biological applications, with a typical dimension in the 5–15 μm range.

This analysis gives useful indications for the design of micro-optical elements in FLICE fabrication. Further analysis could include the characterization of sub-micrometric roughness using AFM or an optical profilometer and evaluation of the thermal annealing effect on more advanced optical elements, such as embedded lenses.

## 4. Materials and Methods

### 4.1. FLM Fabrication: Setup and Parameters

**Figure 4:**
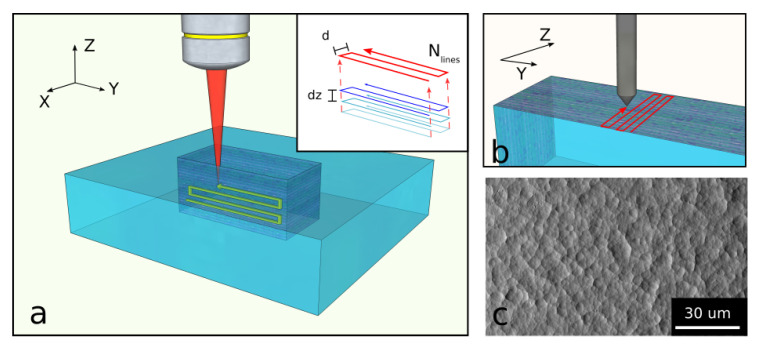
Schemes of the surface fabrication and analysis: (**a**) laser irradiation pattern geometry; (**b**) stylus profilometer raster scan analysis; (**c**) SEM image of a FLICE (femtosecond laser irradiation followed by chemical etching) machined surface.

The fs-laser source used for laser writing consisted of a commercial Ytterbium-based laser (Spirit 1040-16, Spectra-Physics, Stahnsdorf, Germany) emitting at 1042 nm working at 1 MHz repetition rate and with a pulse duration of approximately ≈400 fs. An external LBO crystal was used for second harmonic (SH) generation, obtaining a green light at 521 nm. The laser SH was focused from the top of the sample using a 50 × 0.6 NA objective (Zeiss, Jena, Germany). The linear laser polarization direction can be adjusted using a half-wave plate placed in the SH generation stage. The sample was mounted on a three-axes motorized stage (FIBERGlide 3D, Aereotech Inc., Pittsburgh, PA, USA). We realized all samples in JG1 fused silica windows of 1 mm thickness (Focktech Photonics, Inc., Fujian, China). The surfaces for the roughness analysis were realized by irradiating 4 slots along the Z direction, i.e., the laser propagation axis (see Figure 4a) in order to realize a parallelepiped that could be then detached from the bulk and separately analyzed. The irradiation pattern of the single surface, shown in the inset of Figure 4a, consisted in several XY planes stacked along the Z direction. Each plane was composed of a variable number of laser lines $N_{lines}$ (from 1 to 6) spaced d=1μm each. Both the vertical spacing of the planes (*dz*) and the number of lines per plane were changed from sample to sample to study its impact on the residual roughness. The surfaces had a height of 1 mm (corresponding to the glass thickness) and a width of 2 mm. We used a fixed translation speed of 1.5 mm/s and a fixed pulse energy of 210 nJ, parameters chosen to guarantee nanograting formation at all depths in the sample. The chemical etching was performed using a hydrofluoric acid and deionized water solution at 20% volume concentration. The acid was kept at a constant temperature of $35{\;}^{\circ }$C and we used an ultrasound bath to favor acid diffusion inside the etched slots. The samples were immersed in acid for approximately 1 h.

The embedded mirrors were realized with the same procedure as that of the single surfaces. Moreover, the external perimeter of the device was realized at the same time in order to guarantee perfect alignment between the mirror and the device facets.

### 4.2. Thermal Smoothing

**Figure 5:**
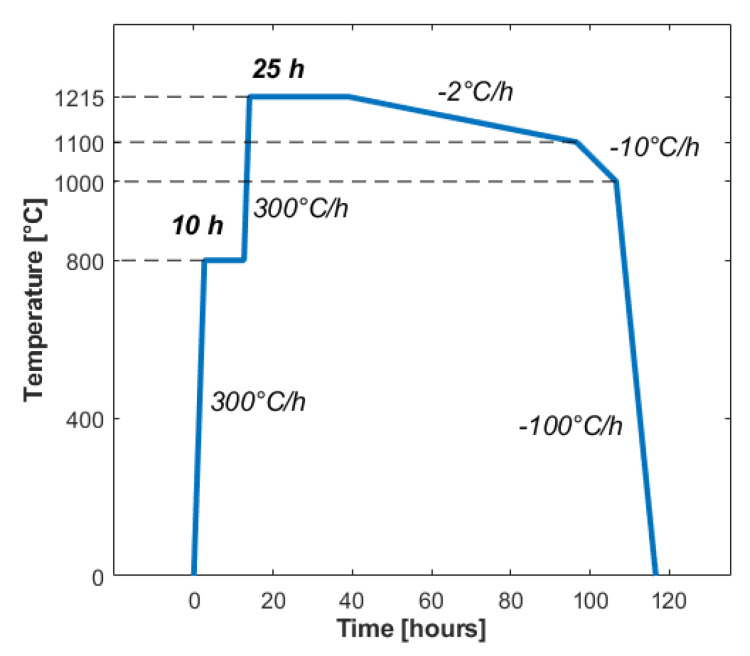
Thermal annealing procedure parameters.

For thermal isotropic annealing of the surfaces, we used a furnace (Nabertherm GmbH, Lilienthal, Germany L5/13/P330) with no controlled atmosphere. Before the treatment, the samples were placed overnight in a solution of sulphuric acid and potassium dichromate and then rinsed in deinonized water to remove surface contamination. The glass parallelepipeds were placed in the center of the furnace, with the surface of interest placed on the top, to avoid contamination from contact with the furnace floor. The procedure consisted of a first heating of the sample up to $1215{\;}^{\circ }$C, with a step at $800{\;}^{\circ }$C for 10 h. The furnace was maintained at maximum temperature for 25 h, before slowly cooling it down to $1000{\;}^{\circ }$C. The sample was then cooled down to room temperature with a fast ramp of $-100{\;}^{\circ }$C/h. The details are reported in Figure 5.

### 4.3. Roughness Analysis

The treated surfaces have been firstly observed with brightfield transmission microscope and SEM (Phenomenon Pro, Thermo Fisher Scientific Inc., Waltham, MA, USA), looking for major surface defects or the presence of cracks. Then a quantitative analysis was performed using a stylus profilometer (Tencor P-17, KLA Corporation, Milpitas, CA, USA). The instrument allowed us to inspect a surface of 100μm by 100μm in approximately 25 min, with a lateral resolution of 1μm and a minimum detectable Z displacement of 0.2 nm. For consistency between measures, we always analyzed a squared area at 200μm distance from the inferior facet of the parallelepiped. The measurement was carried out in raster scan mode, i.e., moving the stylus in contact with the surface along parallel lines, as reported in the scheme in Figure 4b. A typical SEM-acquired image is reported in Figure 4c, showing the texture of the surface under analysis. Lo Turco et al. [21] showed how FLICE machined surface characteristics depend not only on the roughness average value but also on its spatial distribution. For this reason, in our study, we focused on two main parameters: the root mean square (S${}_{q}$ or RMS) roughness and the 2D power spectral density (PSD${}^{2D}$). The first is a standard quantification of the average value of the surface height profile, and it is also the standard deviation of the heights. It can be computed as follows:

(1) $$S_{q}=\sqrt{\frac{1}{A}{\;\int \int \;}_{A}Z^{2}\left(x,y\right)\;dxdy}$$

where A is the total analyzed area in the xy plane and Z(x,y) is the surface heights profile [32]. The PSD${}^{2D}$ provides a representation of the surface roughness as a function of its spatial frequencies, i.e., the inverse of the spatial wavelengths. It can be computed as

(2) $$PSD^{2D}\left(q_{x},q_{y}\right)=\frac{1}{A}\;|\tilde{Z}\left(q_{x},q_{y}\right){|}^{2}$$

where $\tilde{Z}$ is the discrete Fourier transform of the discrete set of data Z(x,y), and $q_{x}$ and $q_{y}$ are the spatial frequencies defined as $q_{i}=2\pi *L_{i}/N_{i}$, with $N_{i}$ number of equispaced acquired point in the i direction and with $L_{i}$ total length along the i direction. The PSD${}^{2D}$ of a finite and non-periodic set of data was computed including a Welch window function to smooth the frequency contribution given by the edges of the dataset.

The data were acquired and filtered using the APEX 3D software (KLA Corporation, Milpitas, CA, USA) form-removal function in order to virtually remove the form profile of the surface and to limit the analysis to the proper roughness. All analyses were carried out using custom-made MATLAB (MathWorks) codes. The effects of surface roughness were characterized by looking at the performances of embedded mirrors. The outer edges of the samples were mechanically polished in order to minimize their impact on the light transmission. A USAF-1951 target (Thorlabs GmbH, 85232 Bergkirchen, Germany) was shone with collimated light coming from a microscope lamp. The obtained image was then reflected by the mirrors and projected by a microscope objective (10× 0.25 NA) onto a CMOS camera (Edmund Optics, Barrington, NJ, USA). The acquired images were then analyzed using Fiji (ImageJ, NIH, Bethesda, MD, USA) application to determine the maximum level of resolution achievable.

## Figures and Tables

**Table 1:** Sample fabrication parameters and measured root mean squared (RMS) roughness before thermal annealing ($S_{q}$). *Pol* indicates the polarization orientation with respect to the line writing direction, and *dz* is the spacing between horizontal planes.

Sample	N${}_{\mathrm{lines}}$	dz	Pol	$S_{q}$
A	6 lines	2 μm	⊥	43 nm
B	single line	2 μm	⊥	57 nm
C	6 lines	5 μm	⊥	28 nm
D	6 lines	10 μm	⊥	35 nm
E	6 lines	2 μm	‖	63 nm

## Data Availability

All data needed to support the conclusions of the paper are present in the text. Raw data are available upon request.
